# The prognostic significance of nuclear DNA content in invasive breast cancer--a study with long-term follow-up.

**DOI:** 10.1038/bjc.1989.342

**Published:** 1989-11

**Authors:** S. Toikkanen, H. Joensuu, P. Klemi

**Affiliations:** Department of Pathology, University of Turku, Finland.

## Abstract

The nuclear DNA content of 351 breast carcinomas was determined by flow cytometry from paraffin-embedded tissue to assess the prognostic significance of DNA ploidy, the DNA index (DI) and the S-phase fraction (SPF). The minimum follow-up of the patients was 22 years, and they were all from a defined urban population. DNA ploidy correlated with histological type and grade, mitotic count and nuclear pleomorphism (P less than 0.0001), and also with axillary nodal status (P = 0.0005), tumour necrosis (P = 0.001), primary tumour size (P = 0.03), menopausal status (P = 0.004) and the presence of distant metastases at the time of the diagnosis (P = 0.04). Survival corrected for intercurrent deaths of the patients with a diploid tumour was better than that of the patients with a non-diploid tumour (P = 0.0001, 48% vs 28% at 25 years). SPF had prognostic significance in both axillary node positive and negative patients, but ploidy and DI only in the node negative group, and their significance was greater in post-menopausal than in premenopausal patients. Axillary nodal status, primary tumour size, histological grade and the type of tumour margin circumscription were the most important independent prognostic factors in Cox's multivariate analysis, and SPF had independent prognostic value, whereas ploidy and DI did not. It is concluded that DNA ploidy, DI and SPF have long-term prognostic significance in breast cancer.


*Br. J. Cancer* (1989), **60**, 693–700
© The Macmillan Press Ltd., 1989

### The prognostic significance of nuclear DNA content in invasive breast cancer – a study with long-term follow-up

S. Toikkanen[1], H. Joensuu[2] & P. Klemi[1]

*Department of [1]Pathology, and [2]Radiotherapy, University of Turku, Kiinamyllynk 8–10, SF-20520 Turku, Finland.*

**Summary** The nuclear DNA content of 351 breast carcinomas was determined by flow cytometry from paraffin-embedded tissue to assess the prognostic significance of DNA ploidy, the DNA index (DI) and the S-phase fraction (SPF). The minimum follow-up of the patients was 22 years, and they were all from a defined urban population. DNA ploidy correlated with histological type and grade, mitotic count and nuclear pleomorphism ($P<0.0001$), and also with axillary nodal status ($P=0.0005$), tumour necrosis ($P=0.001$), primary tumour size ($P=0.03$), menopausal status ($P=0.004$) and the presence of distant metastases at the time of the diagnosis ($P=0.04$). Survival corrected for intercurrent deaths of the patients with a diploid tumour was better than that of the patients with a non-diploid tumour ($P=0.0001$, 48% *vs* 28% at 25 years). SPF had prognostic significance in both axillary node positive and negative patients, but ploidy and DI only in the node negative group, and their significance was greater in post-menopausal than in premenopausal patients. Axillary nodal status, primary tumour size, histological grade and the type of tumour margin circumscription were the most important independent prognostic factors in Cox's multivariate analysis, and SPF had independent prognostic value, whereas ploidy and DI did not. It is concluded that DNA ploidy, DI and SPF have long-term prognostic significance in breast cancer.

The need of reliable prognostic factors in breast cancer has increased, since recent advances in treatment, such as adjuvant hormonal and chemotherapy, require their careful assessment. Breast cancer is biologically a heterogeneous disease, and the prediction of its clinical outcome is difficult in individual cases. A number of factors, such as the clinical stage, nodal status, primary tumour size, histological type, histological and cytological grade of differentiation, mitotic count or index, steroid receptor content, presence of tumour necrosis, immunohistochemical staining properties and amplification of oncogenes have been found to be of prognostic value (Fisher *et al.*, 1984; Baak *et al.*, 1985; Shek & Godolphin, 1988; Kuhajda & Eggleston, 1985; Leatham & Brooks, 1987; Slamon *et al.*, 1987).

The nuclear DNA content determined by flow cytometry has recently emerged as a new prognostic factor in breast cancer. The technique has gained some popularity, since flow cytometric histograms are rapid to produce and often easy to interpret. It has been suggested that breast carcinomas with an abnormal (non-diploid or aneuploid) nuclear DNA content are associated with less favourable prognosis than carcinomas with a normal (diploid) DNA content (Hedley *et al.*, 1984, 1987; Coulson *et al.*, 1984; Ewers *et al.*, 1984; Thorud *et al.*, 1986; Baildam *et al.*, 1987; Cornelisse *et al.*, 1987; Kallioniemi *et al.*, 1987; Stål *et al.*, 1989). Similarly, the DNA index (DI, the relative DNA content of an aneuploid stemline of cells as compared with diploid cells) and the percentage of S-phase cells in the DNA histogram (S-phase fraction, SPF) have been found by some to be of prognostic significance (Dowle *et al.*, 1987; Hedley *et al.*, 1987; Kallioniemi *et al.*, 1988; Stål *et al.*, 1989; McDivitt *et al.*, 1986; Klintenberg *et al.*, 1986). However, the prognostic value of nuclear DNA content analysis is still unsettled. Some authors report DNA content to be the most important prognostic variable in breast cancer, and to have independent prognostic value in a multivariate analysis (Kallioniemi *et al.*, 1988), whereas others find it to have prognostic value only in a univariate analysis (Stål *et al.*, 1989; Hedley *et al.*, 1984), and yet a few fail to find any prognostic significance at all when overall survival is concerned (van der Linden *et al.*, 1989; Uyterlinde *et al.*, 1988; Owainati *et al.*, 1987). The series published have often been heterogeneous, comparison of ploidy with other prognostic variables selective and inadequate, and the follow-up times short.

Correspondence: S. Toikkanen.
Received 13 April 1989; and in revised form 21 June 1989.

The purpose of the present study was to investigate the significance of DNA ploidy, DI and SPF on the long-term prognosis of breast cancer in a defined urban population, and their correlation to a number of suggested clinicopathological prognostic variables.

### Materials and methods

### *Patients*

Four hundred and sixty-one cases of histologically verified female breast carcinoma were diagnosed in the city of Turku in South-Western Finland from 1945 to 1965 according to hospital records and data from the Finnish Cancer Registry. During these years the female population increased from 48,100 to 74,800. The age-adjusted incidence figures for breast cancer are available since 1953, and from the 5-year period 1953–57 to 1963–67 the incidence increased from 30.8 to 41.6 per 100,000 women. The great majority of the patients were treated at the University Central Hospital of Turku, and the rest at the City Hospitals. Seven patients were treated elsewhere, and five were lost from follow-up, and these cases were excluded from the study.

Paraffin blocks for DNA content determination were available in 430 cases with complete follow-up data, and in 384 cases (89%) an interpretable DNA histogram was obtained. Thirty-three of the 384 patients were excluded, because 20 patients developed a second primary carcinoma in the remaining breast, and 13 had either intraductal *in situ* carcinoma or Paget's disease, leaving 351 patients with invasive carcinoma in the series. Two patients who died within 1 month from the diagnosis were not included in the survival analyses ($n=349$).

The clinical data and cause of death were obtained from the hospital records, which were reviewed, and from the files of the Finnish Cancer Registry, the Central Statistical Office of Finland, and from local authorities. All autopsy protocols and histological sections were reviewed. The mean and median age at diagnosis were 56 years (range 30–89 years), and patients older than 49 years were considered as post-menopausal. The mean follow-up time was 28 years (median, 27 years, range 22–42 years). Thirty-five (10%) of the patients had stage I, 190 (54%) stage II, 103 (29%) stage III, and 23 (7%) stage IV cancer according to the UICC post-surgical TNM classification. Thirteen patients had inflammatory and 16 ulcerative cancer. Two-hundred and three (58%) patients were treated with radical mastectomy, 75 with mastectomy and axillary evacuation, 54 with simple mastectomy, 15 with simple excision and four had biopsy only. Postopreative radiotherapy was given to 243 (69%) patients.

### *Histology*

New Haematoxylin & Eosin and van Gieson stained slides were prepared, and the original van Gieson stained slides were reviewed in each case. All available biopsy material before and after cancer diagnosis was reviewed. The histological grading and typing of the tumours was done, slightly modifying the WHO classification. The tumours were subsequently grouped in univariate and multivariate analyses into three types; (1) infiltrating ductogenic carcinoma not otherwise specified (NOS, includes also apocrine, mixed mucinous, and atypical medullary types); (2) infiltrating lobular carcinoma (includes variants); and (3) other special types (includes tubular, medullary, cribriform, papillary, metaplastic and pure mucinous carcinomas). Several other histopathological features, including the number of mitoses per high power field, tubule formation, nuclear pleomorphism, extent of tumour necrosis, amount of stromal fibrosis and elastin, inflammatory cell reaction in and around the tumour, type of tumour margin circumscription (definite margin *vs* diffuse growth pattern) and extent of intraductal growth were evaluated semiquantitatively.

### *DNA flow cytometry*

Paraffin-embedded biopsies were processed for flow cytometry by the method of Hedley *et al.* (1983) with slight modifications. Two 50 μm sections were cut, and one or more adjacent 5 μm control sections were cut for light microscopy. DNA was stained with propidium iodide (Vindeløv *et al.*, 1983), and flow cytometry was done with a FacStar flow cytometer (Becton-Dickinson Immunocytometry Systems, Mountain View, CA). A 488 nm argon laser line run at 600 mW was used for fluorescence excitation. A 585 ± 42 nm band-pass filter was used in front of the red photomultiplier to block the laser light. For each histogram 20,000 particles were analysed.

DNA ploidy was independently assessed by two of the authors without any knowledge of the clinicopathological or survival data. Histograms with a symmetrical G0/G1 peak were classified as diploid, and those with an asymmetrical G0/G1 peak (with a 'shoulder') as near-diploid. Because there was no difference in survival between patients with a diploid or near-diploid tumour, they were combined in statistical analyses and formed the 'diploid' subgroup. If two G0/G1 peaks were present, the histogram was classified as aneuploid and, if more than two, as multiploid. A histogram with a G0/G1 peak at 4N and a G2/M peak at 8N was classified as tretraploid. Because there was no difference in survival between patients with aneuploid, tetraploid or multiploid cancer, there were combined in statistical analyses and formed the 'non-diploid' subgroup. Examples of different types of DNA histograms are given in Figure 1. The DNA index (DI) was calculated by dividing the modal channel number of an aneuploid peak by the modal channel number of the diploid peak. The peak with the least DNA content was taken as the diploid peak. The coefficient of variation (CV) ranged from 2.6 to 9.8. Diploid tumours with CV over 10% and those with excessive cell debris were not included in the study. The S-phase fraction (SPF) was calculated according to the rectilinear method of Baisch *et al.* (1975). SPF could be calculated in 223 (64%) cases. It was not calculated if the height of the S-phase could not be reliably assessed because of overlapping stemlines or the presence of cell debris. The height of SPF was measured near the G2/M peak to avoid counting cell debris. In aneuploid cases with a large DI (> 1.3), SPF was calculated for the aneuploid stemline only.

### *Statistical methods*

Frequency tables were analysed with the $\chi^2$ test. Comparison of age at the diagnosis and SPF in different ploidy groups was done with Kruskal–Wallis' analysis of variance and Mann–Whitney's *U* test because of the markedly non-normal distributions. The survival analysis was performed with the BMDP computer program (BMDP Statistical Software, Department of Biomathematics, University of California, Los Angeles, CA). The cumulative survival was estimated with the product-limit method, and comparison of survival between groups was calculated by Wilcoxon–Breslow and Mantel–Cox statistics. Both crude survival and survival corrected for intercurrent deaths were calculated. The relative importance of prognostic factors was assessed with Cox's

**Figure 1** Examples of DNA flow cytometric histograms. **a**, A diploid histogram with one roughly symmetric G1 peak. SPF 4.1% **b**, A near diploid histogram with an asymmetric G1 peak. SPF 3.7%. **c**, An aneuploid histogram with two G1 peaks. DI 1.73, SPF 16.1%. **d**, An aneuploid histogram with DI 1.19, SPF 6.1%. **e**, A tetraploid histogram with a tetraploid peak on the channel 118, and the diploid peak on channel 60. SPF 23.7%. **f**, A multiploid histogram with two aneuploid peaks (DI 1.67 and 1.88). d = diploid stemline, a = aneuploid stemline, t = tetraploid stemline, s = S-phase fraction.

proportional hazard model (BMDP 2L). All $P$ values are two-tailed.

### Results

### *DNA ploidy*

One hundred and eleven (32%) of the cancers were diploid (74 had a symmetrical and 37 as asymmetrical G1 peaks), and 240 (68%) non-diploid (157 were aneuploid, 27 tetraploid and 56 multiploid). The distribution of DNA indices is shown in Figure 2. The distribution is bimodal with peaks in the diploid/near diploid region, and in the hypertriploid/tetraploid region. SPF ranged from 2.0 to 38.0%, median 9.0% (mean 11.6%, s.d. 7.9%).

### *Survival*

The crude survival of all patients was 34% 10 years and 17% 25 years after the diagnosis, and the corresponding figures corrected for intercurrent deaths were 41% and 34%. Corrected survival of patients with diploid carcinoma was more favourable than that of the patients with non-diploid cancer ($P = 0.0001$). The 10-year survival of the patients with diploid cancer was 53%, and the 25-year survival 48%, whereas the corresponding figures for non-diploid cancer were 36% and 28% (Figure 3). There was no significant difference in survival between patients with aneuploid, tetraploid, or multiploid cancer.

After a series of calculations, the DI value 1.3 was found to have most prognostic significance as a cut-off value. Carcinomas with $DI < 1.3$ had more favourable prognosis than those with $DI > 1.3$ ($P < 0.0001$, Figure 3). Carcinomas with $> 7\%$ S-phase cells were associated with inferior survival as compared with those with SPF $< 7\%$ ($P < 0.0001$ (Figure 4); the cut-off point was found by serial calculations with different SPF values). If the cases in which SPF was calculated for the aneuploid stemline only were considered ($DI > 1.3$, $n = 98$), carcinomas with $SPF > 13\%$ had inferior prognosis ($P = 0.04$), and if the rest of the cases were analysed ($DI < 1.3$, $n = 124$), the best cut-off value was again 7% ($P = 0.0007$).

The prognostic influence of ploidy, DI and SPF after stratification according to the menopausal or axillary nodal status is shown in Table I. DNA ploidy and DI did not have prognostic significance in the axillary nodes positive group, whereas SPF was a significant prognostic factor in all patient groups studied.

### *Correlations with other prognostic factors*

Correlation of DNA ploidy, DI and SPF with several clinicopathological prognostic factors is shown in Tables II and III. They show a strong correlation with many other factors, such as histological type and grade, tubule formation, number of mitoses, nuclear pleomorphism, the primary tumour size, extent of tumour necrosis, the presence of elastin, axillary nodal status and the presence of distant metastases at the time of the diagnosis.

Non-diploid DNA content was also associated with age at diagnosis, 74% of the post-menopausal and 59% of the premenopausal patients had non-diploid cancer ($P = 0.004$). The mean age at diagnosis of the patients with a non-diploid cancer was 56.2 years (s.d., 11.0 years), as compared with 53.9 years (s.d., 12.8 years) in patients with diploid cancer ($P = 0.03$). The patients with tetraploid cancer were older than those with aneuploid cancer at diagnosis ($P = 0.006$, the mean age of the tetraploid group was 62.4 years, and that of the aneuploid group 55.7 years), but the mean age of the patients with multiploid cancer and those with aneuploid cancer did not differ ($P = 0.7$).

DNA non-diploidy was associated with the clinical stage: 49%, 67%, 73% and 87% of the stage I, II, III and IV tumours, respectively, were non-diploid ($P = 0.01$). Only 21

**Figure 2** Distribution of the DNA indices in 351 cases of breast carcinoma. The DI of the diploid cancers is 1.00, of the near diploid 1.05 and of the non-diploid >1.05.

**Figure 3** Survival corrected for intercurrent deaths by DNA ploidy and DNA index. The number of patients at risk is given in brackets. $P$ values are given according to the Wilcoxon–Breslow/Mantel–Cox tests.

**Figure 4** Corrected survival by the S-phase fraction (SPF). $P$ values are given according to the Wilcoxon–Breslow/Mantel–Cox tests.

(17%) of the 123 patients with non-diploid cancer had SPF $< 7\%$ as compared with 72 (72%) of the diploid cancers ($P < 0.0001$). The median SPF of non-diploid tumours was 15.0% (mean 15.4%; S.D., 8.3%) as compared with 6.0% (mean 6.8%; s.d., 3.5%) in diploid tumours ($P = 0.0001$).

No association of ploidy, DI or SPF could be found with the extent of tumour stromal fibrosis, type of tumour margin, presence of inflammatory carcinoma or skin ulceration, mixed histology of the primary tumor, localisation of carcinoma in the breast, laterality between the left and the right breast, duration of symptoms, family history of breast cancer or occurrence of a second primary cancer in other organs during the follow-up. However, carcinomas with DI < 1.3 were more often associated with severe inflammatory cell reaction than carcinomas with DI > 1.3 ($P = 0.008$), and also non-diploid carcinomas had such a tendency ($P = 0.08$). Carcinomas with SPF < 7% were more often associated with principal intraductal growth pattern than those with SPF > 7% ($P = 0.05$).

**Table I** Influence of DNA ploidy, DNA index and S-phase fraction on 25-year corrected survival when patients with breast cancer were stratified according to menopausal and axillary nodal status

| | *Patient stratification* | | | |
|---|---|---|---|---|
| | *Premenopausal* $P_w/P_m$ | *Post-menopausal* $P_w/P_m$ | $N_0$ $P_w/P_m$ | $N_{1-3}$ $P_w/P_m$ |
| DNA ploidy diploid *vs.* non-diploid | 0.09/0.07 $n = 124$ | 0.0006/0.001 $n = 225$ | 0.008/0.01 $n = 158$ | 0.28/0.35 $n = 191$ |
| DNA index ≤1.30 *vs.* >1.30 | 0.02/0.009 $n = 124$ | 0.003/0.005 $n = 225$ | 0.001/0.003 $n = 158$ | 0.42/0.35 $n = 191$ |
| S-phase fraction ≤7% *vs.* >7% | 0.004/0.001 $n = 78$ | 0.0001/0.001 $n = 144$ | 0.01/0.02 $n = 104$ | 0.0001/0.001 $n = 118$ |

Significance of the difference in survival is given according to a Wilcoxon–Breslow test ($P_w$) which gives greater weight to early observations, and according to a Mantel–Cox test ($P_m$), which gives equal weight to all observations.

**Table II** Clinicopathological features in 351 cases of breast cancer and their relation to DNA ploidy and the DN index (DI)

| *Feature* | *n* | *%* | *Non-diploid* | *%* | *P* | *DI>1.30* | *%* | *P* |
|---|---|---|---|---|---|---|---|---|
| All patients | 351 | | 240 | 68 | | 202 | 58 | |
| Age years | | | | | | | | |
| ≤49 | 124 | 35 | 73 | 59 | 0.004 | 57 | 46 | 0.001 |
| >49 | 227 | 65 | 167 | 74 | | 145 | 64 | |
| Histological type | | | | | | | | |
| ductal | 267 | 76 | 201 | 75 | <0.0001 | 177 | 66 | <0.0001 |
| lobular | 53 | 15 | 23 | 43 | | 13 | 25 | |
| tubular | 10 | 3 | 4 | 40 | | 1 | 10 | |
| medullary | 7 | 2 | 6 | 86 | | 6 | 86 | |
| other types | 14 | 4 | 6 | 43 | | 5 | 36 | |
| Histological grade | | | | | | | | |
| I | 77 | 22 | 26 | 34 | <0.0001 | 15 | 19 | <0.0001 |
| II | 144 | 41 | 104 | 72 | | 85 | 59 | |
| III | 130 | 37 | 110 | 85 | | 102 | 78 | |
| Tubule formation | | | | | | | | |
| extensive or moderate | 94 | 27 | 49 | 49 | 0.0001 | 33 | 35 | <0.0001 |
| slight/none | 257 | 73 | 191 | 74 | | 169 | 65 | |
| Mitoses/HPF[a] | | | | | | | | |
| rare | 120 | 34 | 56 | 47 | <0.0001 | 38 | 32 | <0.0001 |
| 2–3 | 130 | 41 | 96 | 74 | | 81 | 62 | |
| >3 | 101 | 37 | 88 | 87 | | 83 | 82 | |
| Nuclear pleomorphism | | | | | | | | |
| slight | 50 | 14 | 8 | 16 | <0.0001 | 3 | 6 | <0.0001 |
| moderate | 209 | 60 | 157 | 75 | | 130 | 62 | |
| severe | 92 | 26 | 75 | 82 | | 69 | 75 | |
| Necrosis | | | | | | | | |
| none | 204 | 58 | 123 | 60 | 0.001 | 94 | 46 | <0.0001 |
| spotty | 71 | 20 | 55 | 77 | | 52 | 73 | |
| moderate | 48 | 14 | 38 | 79 | | 34 | 71 | |
| severe | 28 | 8 | 24 | 86 | | 22 | 79 | |
| Elastin | | | | | | | | |
| none | 200 | 57 | 148 | 74 | 0.009 | 129 | 65 | 0.002 |
| some to severe | 151 | 43 | 92 | 61 | | 73 | 48 | |
| Tumour size | | | | | | | | |
| $T_1$(≤2 cm) | 39 | 11 | 20 | 51 | 0.03 | 16 | 41 | 0.04 |
| $T_2$(>2–5 cm) | 199 | 57 | 134 | 67 | | 115 | 58 | |
| $T_3$(>5 cm) | 59 | 17 | 43 | 73 | | 33 | 56 | |
| $T_4$ | 54 | 15 | 43 | 80 | | 38 | 70 | |
| Nodal status | | | | | | | | |
| $N_0$ | 158 | 45 | 93 | 59 | 0.0005 | 76 | 48 | 0.002 |
| $N_{1-3}$ | 193 | 55 | 147 | 76 | | 126 | 63 | |
| Distant metastases | | | | | | | | |
| $M_0$ | 328 | 93 | 220 | 67 | 0.04 | 184 | 56 | 0.04 |
| $M_1$ | 23 | 7 | 20 | 87 | | 18 | 78 | |

[a]High power field.

**Table III** Clinicopathological features in 223 cases of breast cancer and their relation to the S-phase fraction (cut-off point 7%)

| *Feature* | *n* | *%* | *SPF>7%* | *%* | *P* |
|---|---|---|---|---|---|
| All patients | 223 | | 130 | 59 | |
| Age years | | | | | |
| $\leqslant$49 | 78 | 35 | 38 | 49 | 0.03 |
| >49 | 145 | 65 | 92 | 63 | |
| Histological type | | | | | |
| ductal | 165 | 74 | 113 | 68 | <0.0001 |
| lobular | 39 | 17 | 11 | 28 | |
| tubular | 5 | 2.5 | 0 | 0 | |
| medullary | 2 | 1 | 2 | 100 | |
| other types | 12 | 5.5 | 4 | 33 | |
| Histological grade | | | | | |
| I | 58 | 26 | 12 | 21 | <0.0001 |
| II | 103 | 46 | 68 | 66 | |
| III | 62 | 28 | 50 | 81 | |
| Tubule formation | | | | | |
| extensive or moderate | 74 | 33 | 31 | 42 | 0.0005 |
| slight/none | 149 | 67 | 99 | 66 | |
| Mitoses/HPF[a] | | | | | |
| rare | 87 | 39 | 28 | 32 | <0.0001 |
| 2–3 | 85 | 38 | 60 | 71 | |
| >3 | 51 | 23 | 42 | 82 | |
| Nuclear pleomorphism | | | | | |
| slight | 41 | 18 | 5 | 12 | <0.0001 |
| moderate | 141 | 64 | 92 | 65 | |
| severe | 41 | 18 | 33 | 80 | |
| Necrosis | | | | | |
| none | 138 | 62 | 67 | 49 | 0.002 |
| spotty | 42 | 19 | 30 | 71 | |
| moderate | 29 | 12 | 22 | 76 | |
| severe | 14 | 7 | 11 | 79 | |
| Elastin | | | | | |
| none | 124 | 56 | 81 | 65 | 0.01 |
| some to severe | 99 | 44 | 49 | 49 | |
| Tumour size | | | | | |
| $T_1$ ($\leqslant$2 cm) | 24 | 11 | 9 | 38 | 0.08 |
| $T_2$ (>2–5 cm) | 127 | 57 | 73 | 57 | |
| $T_3$ (>5 cm) | 38 | 17 | 24 | 63 | |
| $T_4$ | 34 | 15 | 24 | 71 | |
| Nodal status | | | | | |
| $N_0$ | 104 | 47 | 52 | 50 | 0.01 |
| $N_{1-3}$ | 119 | 53 | 78 | 66 | |
| Distant metastases | | | | | |
| $M_0$ | 204 | 91 | 112 | 55 | 0.0008 |
| $M_1$ | 19 | 9 | 18 | 95 | |

[a]High power field.

*Multivariate analyses*

In order to find out the relative importance and independence of DNA ploidy, DI and SPF as prognostic factors, they were tested in Cox's proportional hazard model together with all factors that had prognostic significance ($P<0.05$) as a single factor in univariate analyses.

The results of univariate and Cox's multivariate analyses are shown in Table IV. The most important independent prognostic factor in a multivariate analysis was axillary nodal status ($N_0$ *vs* $N_{1-3}$, $P<0.001$), followed by primary tumour size ($T_1$ *vs* $T_2$ *vs* $T_{3-4}$, $P<0.001$), histological grade (grade I *vs* grade II *vs* grade III, $P<0.001$), type of tumour margin (definite *vs* diffuse, $P<0.001$), extent of tumour necrosis (none to moderate *vs* severe, $P=0.01$), and histological type (other special types *vs* lobular *vs* ductal type, $P=0.02$). DNA ploidy and DI did not appear as independent prognostic variables. However, if SPF was entered in the analysis, it had independent prognostic value ($P=0.02$). Furthermore, slight nuclear pleomorphism, extensive or moderate tubule formation, low number of mitoses, extensive intraductal growth of cancer and age $\leqslant$49 years at diagnosis had favourable impact on prognosis in univariate analyses, but not in a multivariate analysis.

If DNA ploidy, SPF and DI were tested together with the other factors after stratifying the material according to the axillary nodal status, the most important prognostic factor in axillary node negative patients was primary tumour size ($P=0.002$), followed by type of tumour margin ($P=0.01$) and SPF ($P=0.02$); and in axillary node positive patients primary tumour size ($P<0.001$), histological grade ($P=0.001$), type of tumour margin ($P=0.02$), extent of tumour necrosis ($P=0.04$) and SPF ($P=0.07$). If the material was stratified according to the menopausal status, the most important prognostic factors in the premenopausal group were axillary nodal status ($P<0.001$), type of tumour margin ($P=0.01$), mitotic count ($P=0.01$), and tubule formation ($P=0.03$); and in the post-menopausal group axillary nodal status ($P<0.001$), followed by histological grade ($P<0.001$), primary tumour size ($P<0.001$) and type of tumour margin ($P=0.02$).

If crude survival was tested instead of corrected survival in Cox's analysis (and age at diagnosis was excluded from the tested variables), the relative significance of SPF, DI and ploidy was somewhat greater. SPF ($P<0.001$) was the third in rank after axillary nodal status ($P<0.001$) and primary tumour size ($P<0.001$) in relative importance, and the most important single factor ($P=0.002$) in the axillary node

Table IV Prognostic factors in breast cancer, and their influence on 25-year corrected survival, results of univariate and multivariate analyses

| | *Univariate analysis All cases n = 349* | | *Multivariate analysis All cases n = 349* | | *Cases with evaluable SPF n = 222* | |
|---|---|---|---|---|---|---|
| *Factor* | *P* | $\chi^2$ | *P* | *Step*[a] | *P* | *Step*[a] |
| Nodal status | <0.0001 | 135.5 | <0.001 | 1 | <0.001 | 1 |
| Tumour size | <0.0001 | 120.5 | <0.001 | 2 | <0.001 | 2 |
| Histological grade | <0.0001 | 65.8 | <0.001 | 3 | <0.001 | 3 |
| Tumour margin | <0.0001 | 23.0 | <0.001 | 4 | <0.001 | 4 |
| Tumour necrosis | <0.0001 | 34.1 | 0.01 | 5 | n.s. | |
| Histological type | <0.0001 | 34.7 | 0.02 | 6 | n.s. | |
| Nuclear pleomorphism | <0.0001 | 35.5 | 0.08 | 7 | n.s. | |
| Menopausal status | 0.02 | 5.1 | 0.09 | 8 | n.s. | |
| Mitotic count | <0.0001 | 48.9 | n.s. | | n.s. | |
| Tubule formation | <0.0001 | 25.5 | n.s. | | n.s. | |
| S-phase fraction[b] | <0.0001 | 18.6 | – | | 0.02 | 5 |
| DNA index[c] | <0.0001 | 16.6 | n.s. | | n.s. | |
| DNA ploidy | 0.0001 | 14.9 | n.s. | | n.s. | |
| Intraductal growth | 0.02 | 5.5 | n.s. | | n.s. | |

[a]Step of factor removal in Cox's multivariate stepwise analysis. [b]Cut-off point 7%. [c]Cut-off point 1.30. n.s., statistically not significant.

negative group ($n$ = 104). If both SPF and age were excluded from the tested variables, DI with cut-off point 1.3 ($P$<0.001) became the most important variable predicting crude survival in the axillary node negative group ($n$ = 158).

### Discussion

A non-diploid nuclear DNA content, DI value >1.3 and SPF>7% were all correlated with adverse prognosis, and the adverse effect was shown to last for 25 years after the diagnosis. All these variables were associated with many of the known prognostic factors in breast cancer, some of which were more powerful prognostic factors than the ones derived from the DNA content analysis in multivariate analyses.

To our knowledcge, there are no published studies in which DNA content determination has been attempted from paraffin embedded material that has been collected in the 1940 s and 1950 s. Although the number of uninterpretable histograms was the greater the older the sample was (data not shown), the majority of the histograms (89%) were of acceptable quality. The histograms were classified without knowledge of survival or clinicopathological data, and the correlations found were stronger than in many series produced from more recent material (Dressler *et al.*, 1988; Feichter *et al.*, 1988; Haag *et al.*, 1984). The percentage of non-diploid tumours found (68%) is almost identical to the mean percentage of 67% found in 23 studies on breast cancer comprising the total of 5,785 patients (data not shown).

Although DNA flow cytometry has been claimed to be an objective method, the DNA histograms are not always similarly interpreted, resulting in a different percentage of DNA aneuplody found even if the data is similar (Joensuu & Kallioniemi, 1989). The use of DI abolishes some of the problems involved in data interpretation, since near diploid tumours and tumours with a small DI may be grouped together with the diploid tumours. DI is easy to calculate, and was a slightly more powerful prognostic variable than DNA ploidy (Table IV). The great majority of non-diploid tumours have DI>1.3 (Figure 2) and hence both DI>1.3 and DNA non-diploidy are closely related variables (Figure 3).

The SPF value 7% was the most effective cut-off percentage for prognosis. The value of SPF as a prognostic factor is lessened by the fact that it often cannot be reliably assessed, e.g. in cases with overlapping cell populations or presence of cell debris. When SPF of diploid histograms is calculated, a variable number of stromal and non-tumour cells are included usually reducing the relative percentage of SPF, whereas if SPF is calculated from an aneuploid stemline, only cancer cells are considered, and SPF is likely to be higher. In this series only 17% of non-diploid tumours had SPF<7% as compared with 72% of the diploid tumours, which difference may partly be technical. Hence, SPF is also related to DNA ploidy and DI.

Most authors agree that DNA aneuploidy is associated with poor histological grade (Thorud *et al.*, 1986; Moran *et al.*, 1984; Spyratos *et al.*, 1987; Olszewski *et al.*, 1981; Jakobsen *et al.*, 1984; Hedley *et al.*, 1984; McDivitt *et al.*, 1986; Kute *et al.*, 1985; Kallioniemi *et al.*, 1987; Feichter *et al.*, 1988; Dowle *et al.*, 1987), and the mitotic count, nuclear pleomorphism and degree of tubule formation describe much the same thing. The amount of elastin and the extent of necrosis have been associated with histological grade too (Fisher *et al.*, 1984; Kuhajda *et al.*, 1985). The association of ploidy with primary tumour size and axillary nodal status has been controversial (Uyterlinde *et al.*, 1988; Spyratos *et al.*, 1987; Taylor *et al.*, 1983; McDivitt *et al.*, 1986; Cornelisse *et al.*, 1987; Dressler *et al.*, 1988). In this series DNA aneuploidy was significantly more common in large primary tumours, and in tumours with axillary or distant metastases (Table II). It has previously been largely unnoticed that the type of tumour margin is an important prognostic factor in both uni- and multivariate analyses, and this feature has quite unexpectedly no correlation with the DNA analysis derived factors.

The occurrence of aneuploidy in histological subtypes of breast cancer has received scant attention; only mucinous carcinomas have been extensively studied (Toikkanen *et al.*, 1988). The percentage of non-diploid tumours was high in medullary (86%) and ductogenic NOS (74%) carcinomas, and low in lobular (43%) and tubular (40%) carcinomas (Table II). Survival of the patients with lobular carcinoma, or with carcinoma of the other special types, was much better than that of the patients with a ductogenic infiltrating NOS carcinoma ($P$<0.0001). The medullary carcinoma, however, was associated with a favourable outcome despite frequent aneuploidy and, by definition, poor histological differentiation. The good prognosis of medullary carcinoma appears to depend on factors not associated with the ordinary favourable histological features, and it may be associated with its definite circumscription and strong lymphocyte infiltration (Hsu *et al.*, 1981).

As in several other cancers (Joensuu *et al.*, 1986; Klemi *et al.*, 1988), and even in benign tumours (Joensuu & Klemi, 1988), patients with a non-diploid breast tumour were older than those with a diploid tumour (Table II). This has also been noticed by Taylor *et al.* (1983). DNA aneuploid tumours usually have hypertriploid DNA content, and it has been suggested that DNA aneuploidy develops via tetraploidy: tetraploid tumours lose some of their chromosomal material resulting in hypertriploid nuclear DNA content (Ewers *et al.*, 1984). However, patients with tetraploid cancer were older at diagnosis than patients with aneuploid carcinoma ($P = 0.006$), which is poorly compatible with this theory.

As expected, the ability of most of the factors listed in Table IV to predict the final outcome decreased if crude survival was studied instead of survival corrected for intercurrent deaths (*P* values became larger). However, the opposite was true for age at diagnosis, and unexpectedly for DI and SPF. Because special attention was paid to finding the correct cause of death, deaths caused by breast cancer misinterpreted as intercurrent deaths are not a likely explanation for the stronger correlation of DI and SPF with crude than with corrected survival. It is rather a further piece of evidence for the association of advanced age and the tendency to develop non-diploid solid tumours.

Most authors (Thorud *et al.*, 1986; Coulson *et al.*, 1984; Hedley *et al.*, 1987; Kallioniemi *et al.*, 1987; Dowle *et al.*, 1987; Cornelisse *et al.*, 1987; Ewers *et al.*, 1984; Stål *et al.*, 1989), but not all (van der Linden *et al.*, 1989; Uyterlinde et al., 1988; Owainati *et al.*, 1987), agree that DNA aneuploidy is associated with unfavourable survival in breast cancer. In most studies this association has, however, been weak. Cornelisse *et al.* (1987) found only a slight correlation with ploidy and overall survival ($P = 0.04$) despite having the largest histologically verified material published so far ($n = 565$). Contrary to our results, ploidy had no effect on survival in the axillary node negative group, and it had an independent impact on survival in the post-menopausal axillary node positive group. Still others have found ploidy to be significant in a univariate analysis, but not in a multivariate analysis (Hedley *et al.*, 1984; Stål *et al.*, 1989). The most promising results have been published by Kallioniemi *et al.* (1987), who found DNA ploidy to be an independent prognostic factor, even if survival was controlled for nodal status.

The results of different studies must be compared with caution, since differences in histogram analysis, patient materials, follow-up and statistical analyses may be considerable. Unlike in most previous studies, the present series comes from a defined well-documented population, and includes all breast cancers in this area without any selection. The number of clinicopathological factors studied exceeds that of the previous works, which may have significance, because the exclusion of any major prognostic factor from the Cox's analysis may influence the result. However, steroid receptor analyses were not available to us.

It is concluded that DNA ploidy, DI and SPF are important prognostic factors in breast cancer, especially in axillary node negative cancer and in post-menopausal patients, and that they have long-term prognostic influence. However, they are closely associated with other histological and clinical prognostic factors related to cancer morphology, differentiation, tumour size and spread. SPF could be shown to have independent prognostic value in multivariate analyses, but axillary nodal status, size of primary tumour, histological grade and type of tumour margin were more powerful independent prognostic factors. DNA ploidy and DI have prognostic value as single variables in axillary node negative patients, whereas SPF has such value both in axillary node negative and positive patients.

This study was supported by the Cancer Society of Finland. The authors thank the staff of the Finnish Cancer Registry and the local authorities for providing the survival data.

### References

BAAK, J.P.A., VAN DOP, H., KURVER, P.H.J. & HERMANS, J. (1985). The value of morphometry to classic prognosticators in breast cancer. *Cancer*, **56**, 374.

BAILDAM, A.D., ZALOUDIK, J., HOWELL, A. & 5 others (1987). DNA analysis by flow cytometry, response to endocrine treatment and prognosis in advanced carcinoma of the breast. *Br. J. Cancer*, **55**, 553.

BAISCH, H., GÖHDE, W. & LINDEN, W.A. (1975). Analysis of PCP-data to determine the fraction of cells in the various phases of cell cycle. *Radiat. Environ. Biophys.*, **12**, 31.

CORNELISSE, C.J., VAN DE VELDE, C.J.H., CASPERS, R.J.C., MOOLENAAR, A.J. & HERMANS, J. (1987). DNA ploidy and survival in breast cancer patients. *Cytometry*, **8**, 225.

COULSON, P.B., THORNTHWAITE, J.T., WOOLLEY, T.W., SUGARBAKER, E.V. & SECKINGER, D. (1984). Prognostic indicators including DNA histogram type, receptor content, and staging related to human breast cancer patient survival. *Cancer Res.*, **44**, 4187.

DOWLE, C.S., OWAINATI, A., ROBINS, A. & 4 others (1987). Prognostic significance of the DNA content of human breast cancer. *Br. J. Surg.*, **74**, 133.

DRESSLER, L.G., SEAMER, L.C., OWENS, M.A., CLARK, G.M. & MCGUIRE, W.L. (1988). DNA flow cytometry and prognostic factors in 1331 frozen breast cancer specimens. *Cancer*, **61**, 420.

EWERS, S.-B., LÅNGSTRÖM, E., BALDETORP, B. & KILLANDER, D. (1984). Flow-cytometric DNA analysis in primary breast carcinomas and clinicopathological correlations. *Cytometry*, **5**, 408.

FEICHTER, G.E., MUELLER, A., KAUFMANN, M. & 6 others (1988). Correlation of DNA cytometric results and other prognostic factors in primary breast cancer. *Int. J. Cancer*, **41**, 823.

FISHER, E.R., SASS, R. & FISHER, B. (1984). Pathologic findings from the National Surgical Adjuvant Project for Breast Cancers (Protocol No. 4). X. Discriminants for tenth year treatment failure. *Cancer*, **53**, 712.

HAAG, D., GOERTTLER, K. & TSCHAHARGANE, C. (1984). The proliferative index (PI) of human breast cancer as obtained by flow cytometry. *Pathol. Res. Pract.*, **178**, 315.

HEDLEY, D.W., FRIEDLANDER, M.L., TAYLOR, I.W., RUGG, C.A. & MUSGROVE, E.A. (1983). Method for analysis of cellular DNA content of paraffin-embedded pathological material using flow cytometry. *J. Histochem. Cytochem.* **31**, 1333.

HEDLEY, D.W., RUGG, C.A., HG, A.B.P. & TAYLOR, I.W. (1984). Influence of cellular DNA content on disease-free survival of stage II breast cancer patients. *Cancer Res.*, **44**, 5395.

HEDLEY, D.W., RUGG, C.A. & GELBER, R.D. (1987). Association of DNA index and S-phase fraction with prognosis of nodes positive early breast cancer. *Cancer Res.*, **47**, 4729.

HSU, S.U., RAINE, L. & NAYAK, R.N. (1981). Medullary carcinoma of breast: an immunohistochemical study of its lymphoid stroma. *Cancer*, **48**, 1368.

JAKOBSEN, A., POULSEN, H.S., MADSEN, E.L., PETERSEN, S.E. & HANSEN, H.S. (1984). Ploidy level of human breast carcinoma. Relation to histopathologic features and hormone receptor content. *Acta Radiol. Oncol.*, **23**, 103.

JOENSUU, H. & KALLIONIEMI, O.-P. (1989). Different opinions on classification of DNA histograms produced from paraffin-embedded tissue. *Cytometry* (in the press).

JOENSUU, H. & KLEMI, P.J. (1988). DNA aneuploidy in adenomas of the endocrine organs. *Am. J. Pathol.*, **132**, 145.

KALLIONIEMI, O.-P., BLANCO, G., ALAVAIKKO, M. & 4 others (1987). Tumour DNA ploidy as an independent prognostic factor in breast cancer. *Br. J. Cancer.*, **56**, 637.

KALLIONIEMI, O.-P., BLANCO, G., ALAVAIKKO, M. & 5 others. (1988). Improving the prognostic value of DNA flow cytometry in breast cancer by combining DNA index and S-phase fraction. A proposed classification of DNA histograms in breast cancer. *Cancer*, **62**, 2183.

KLEMI, P.J., JOENSUU, H., KIILHOLMA, P. & MÄENPÄÄ, J. (1988). Clinical significance of abnormal nuclear DNA content in serous ovarian tumours. *Cancer*, **62**, 2005.

KLINTENBERG, C., STÅL, O., NORDENSKJÖLD, B., WALLGREN, A., ARVIDSSON, S. & SKOOG, L. (1986). Proliferative index, cytosol estrogen receptor and axillary node status as prognostic predictors in human mammary carcinoma. *Breast Cancer Res. Treat. (Suppl.)*, **7**, 99.

KUHAJDA, F.P. & EGGLESTON, J.C. (1985). Pregnancy-associated plasma protein A and extensive necrosis; clinically significant predictors of early recurrence in stage I estrogen receptor-negative breast carcinoma. *Lab. Invest.*, **53**, 101.

KUTE, T.E., MUSS, H.B., HOPKINS, M., MARSHALL, R., CASE, D. & KAMMIRE, L. (1985). Relationship of flow cytometry results to clinical and steroid receptor status in human breast cancer. *Breast Cancer Res. Treat.*, **6**, 113.

LEATHAM, A.J. & BROOKS, S.A. (1987). Predictive value of lectin binding on breast-cancer recurrence and survival. *Lancet*, **i**, 1054.

VAN DER LINDEN, J.C., LINDEMAN, J., BAAK, J.P.A., MEIJER, C.J.L.M. & HERMAN, C.J. (1989). The multivariate prognostic index and nuclear DNA content are independent prognostic factors in primary breast cancer patients. *Cytometry*, **10**, 56.

MCDIVITT, R.W., STONE, K.R., CRAIG, R.B., PALMER, J.O., MEYER, J.S., & BAUER, W.C. (1986). A proposed classification of breast cancer based on kinetic information. *Cancer*, **57**, 269.

MORAN, R.E., BLACK, M.M., ALPERT, L. & STRAUS, M.J. (1984). Correlation of cell-cycle kinetics, hormone receptors, histopathology, and nodal status in human breast cancer, *Cancer*, **54**, 1586.

OLSZEWSKI, W., DARZYNKIEWICZ, Z., ROSEN, P.P., SCHWARTZ, M.K. & MELAMED, M.R. (1981). Flow cytometry of breast carcinoma: II. Relation of tumor cell cycle distribution to histology and estrogen receptor. *Cancer*, **48**, 985.

OWAINATI, A.A.R., ROBINS, R.A., HINTON, C. & 6 others (1987). Tumor aneuploidy, prognostic parameters and survival in primary breast cancer. *Br. J. Cancer*, **55**, 449.

SHEK, L.L.M. & GODOLPHIN, W. (1988). Model for breast cancer survival; relative prognostic roles of axillary nodal status, TNM stage, estrogen receptor concentration, and tumor necrosis. *Cancer Res.*, **48**, 5565.

SLAMON, D.J., CLARK, G.M., WONG, S.G., LEVIN, W.J., ULLRICH, A. & MCGUIRE, W.L. (1987). Human breast cancer: correlation of relapse and survival with amplification of the HER-2/neu oncogene. *Science*, **235**, 177.

SPYRATOS, F., BRIFFOD, M., GENTILE, A., BRUNET, M., BRAULT, C. & DESPLACES, A. (1987). Flow cytometric study of DNA distribution in cytopunctures of benign and malignant breast lesions. *Anal. Quant. Cytol.*, **9**, 485.

STÅL, O., WINGREN, S., CARSTENSEN, J. & 4 others (1989). Prognostic value of DNA ploidy and S-phase fraction in relation to estrogen receptor content and clinicopathological variables in primary breast cancer. *Eur. J. Cancer Clin. Oncol.*, **25**, 301.

TAYLOR, I.W., MUSGROVE, E.A., FRIEDLANDER, M.L., FOO, M.S. & HEDLEY, D.W. (1983). the influence of age on the DNA ploidy levels of breast tumours. *Eur. J. Cancer Clin. Oncol.*, **19**, 623.

THORUD, E., FOSSÅ, S.D., VAAGE, S. & 4 others (1986). Primary breast cancer. Flow cytometric DNA pattern in relation to clinical and histopathologic characteristics. *Cancer*, **57**, 808.

TOIKKANEN, S., EEROLA, E. & EKFORS, T.O. (1988). Pure and mixed mucinous breast carcinomas: DNA stemline and prognosis. *J. Clin. Pathol.*, **41**, 300.

UYTERLINDE, A.M., SCHIPPER, N.W., BAAK, J.P.A., PETERSE, H. & MATZE, E. (1988). Limited prognostic value of cellular DNA content to classical and morphometrical parameters in invasive ductal breast cancer. *Am. J. Clin. Pathol.*, **89**, 301.

VINDELØV, L.L., CHRISTENSEN, I.J. & NISSEN, N.I. (1983). A detergent-trypsin method for the preparation of nuclei for flow cytometric DNA analysis. *Cytometry*, **3**, 323.


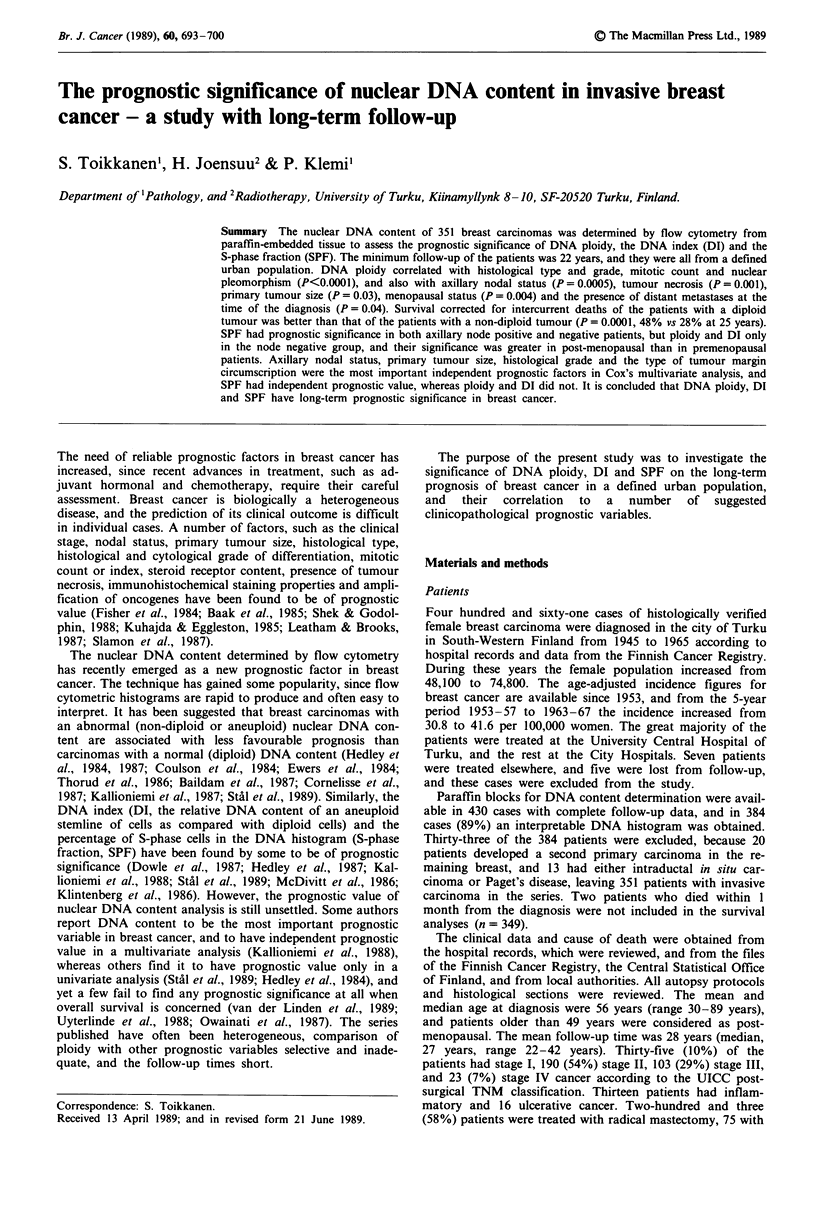

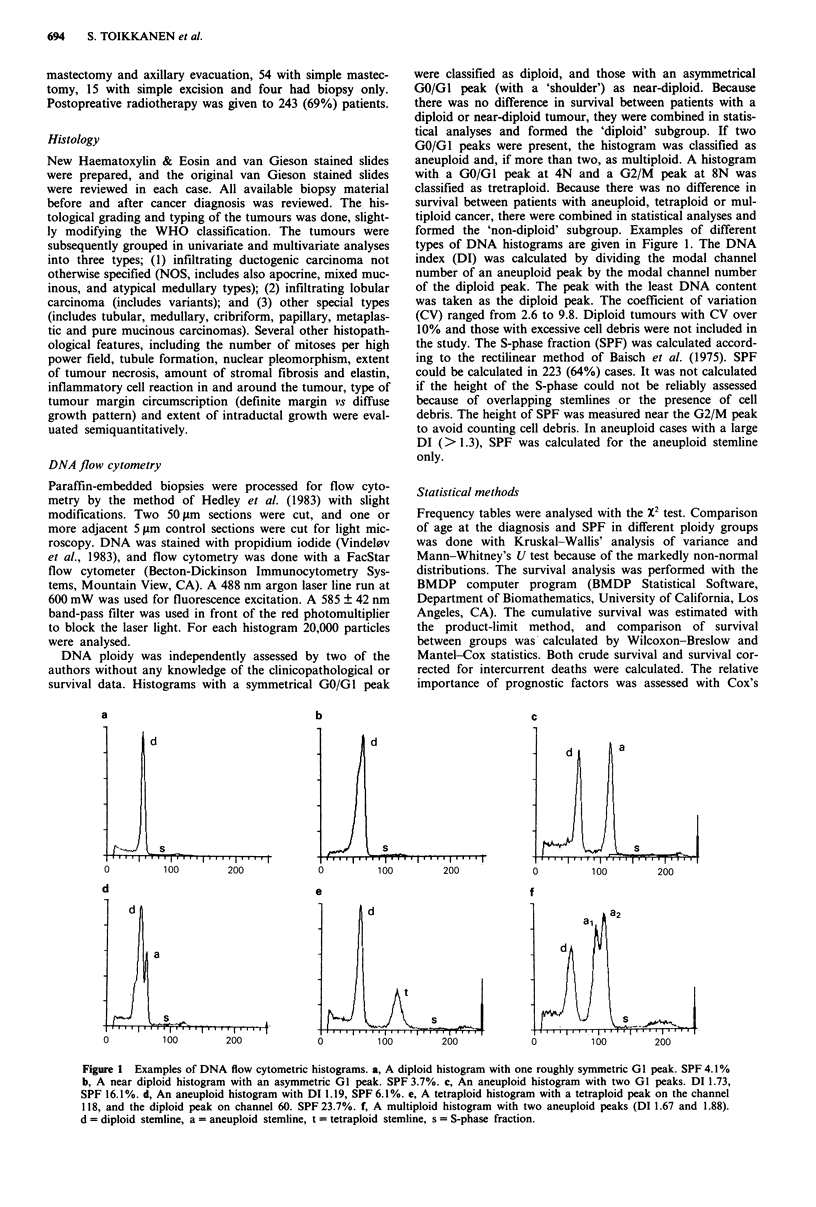

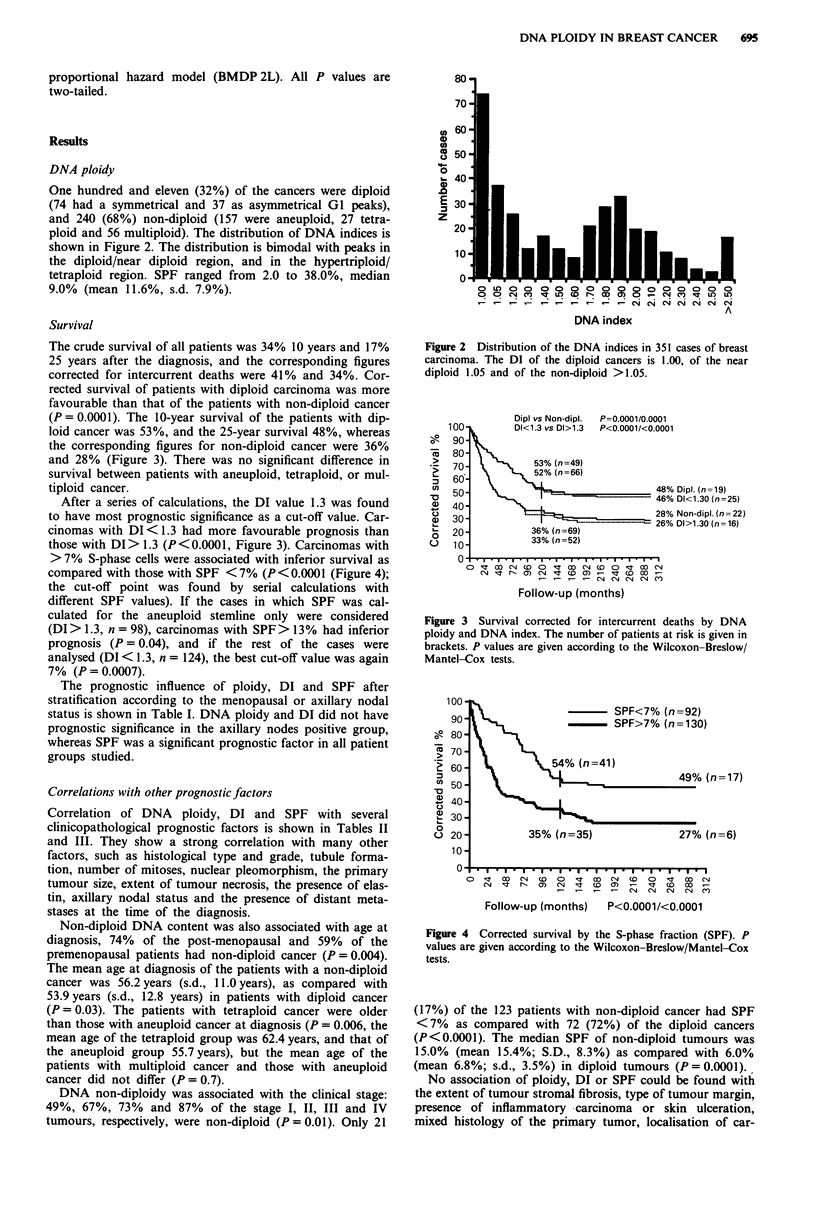

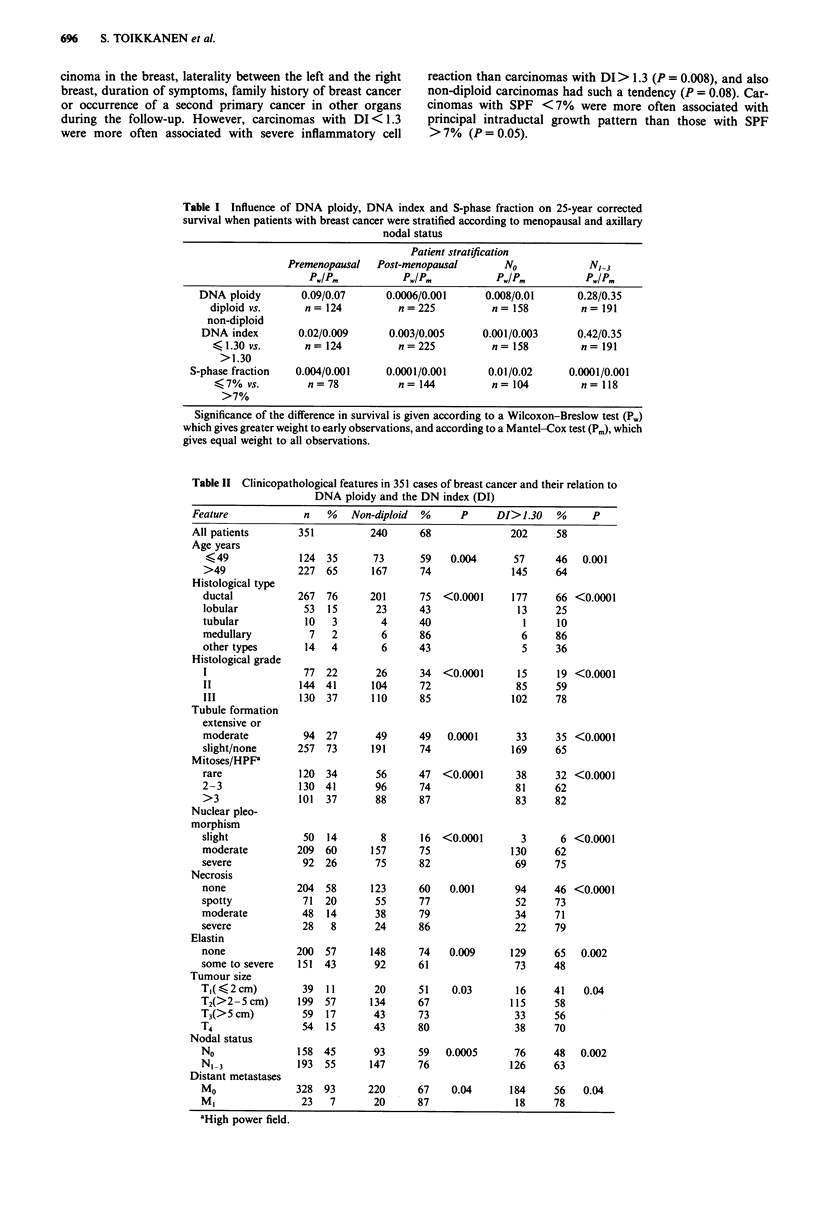

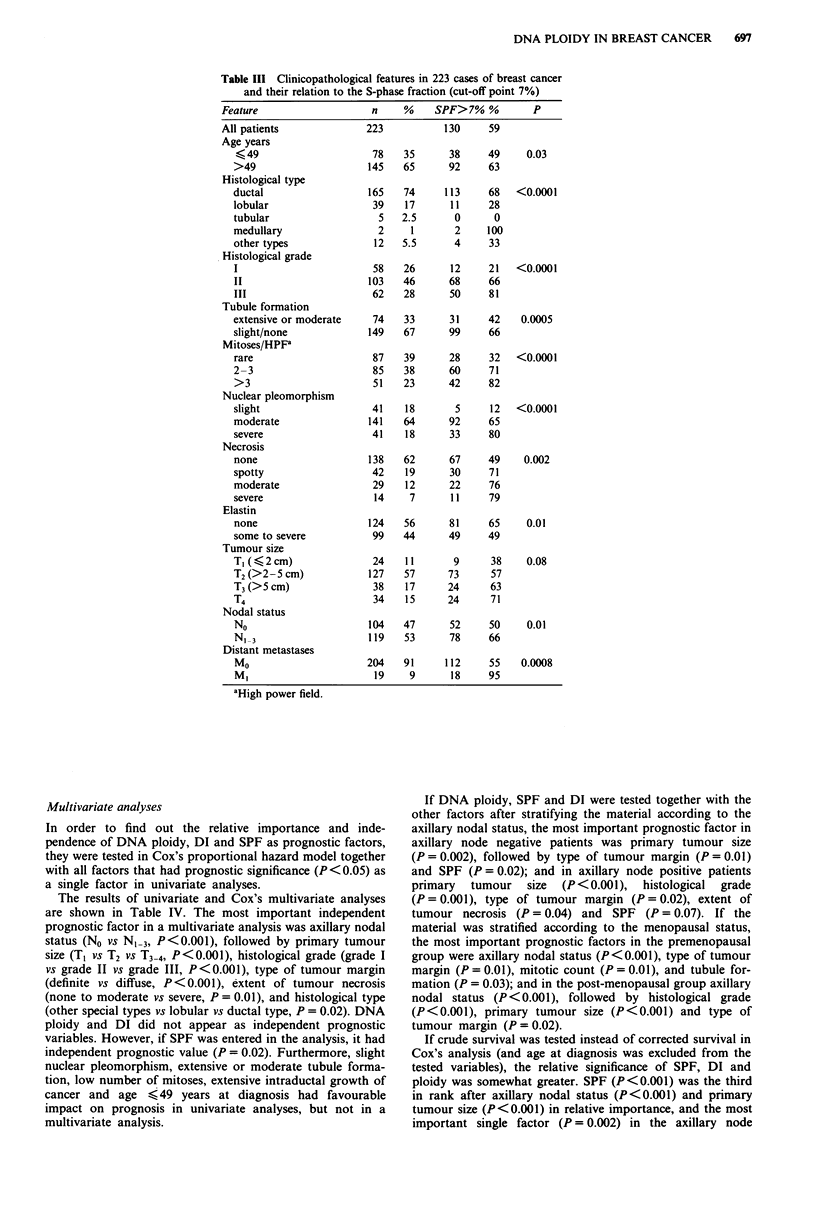

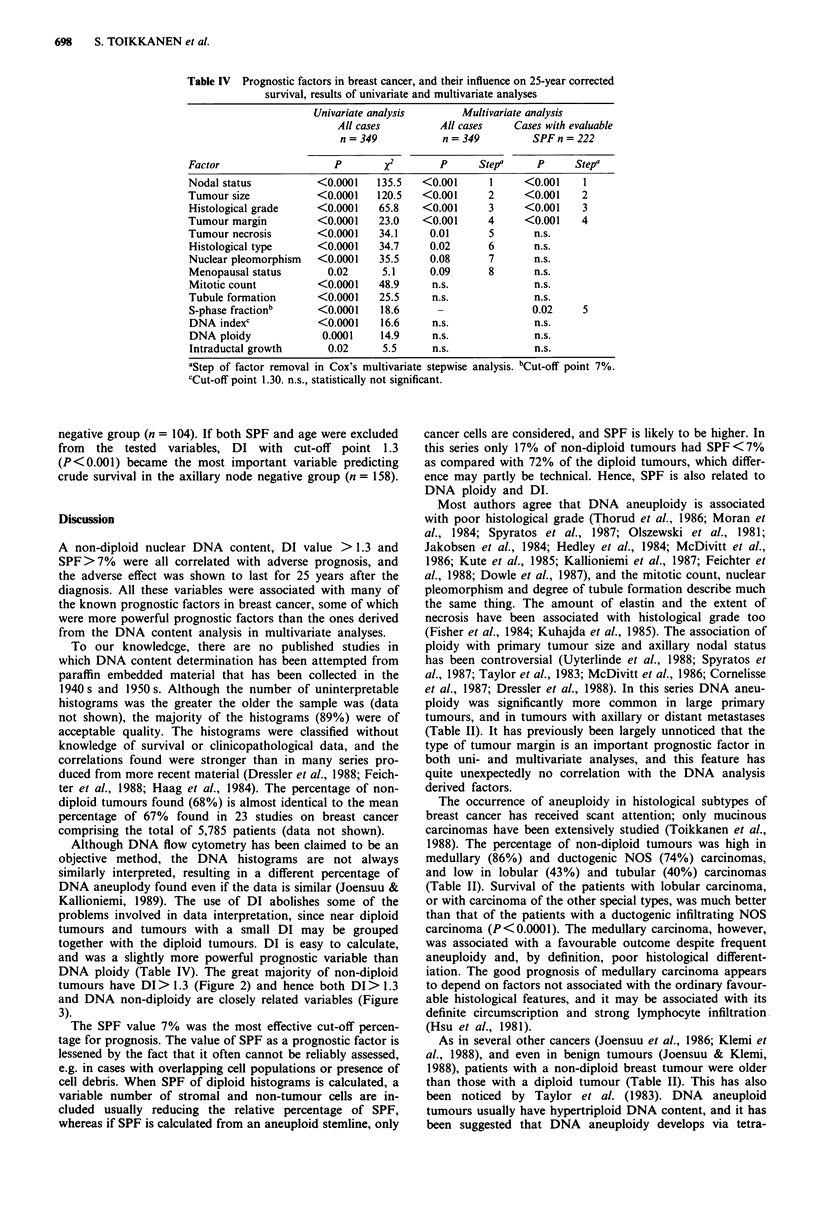

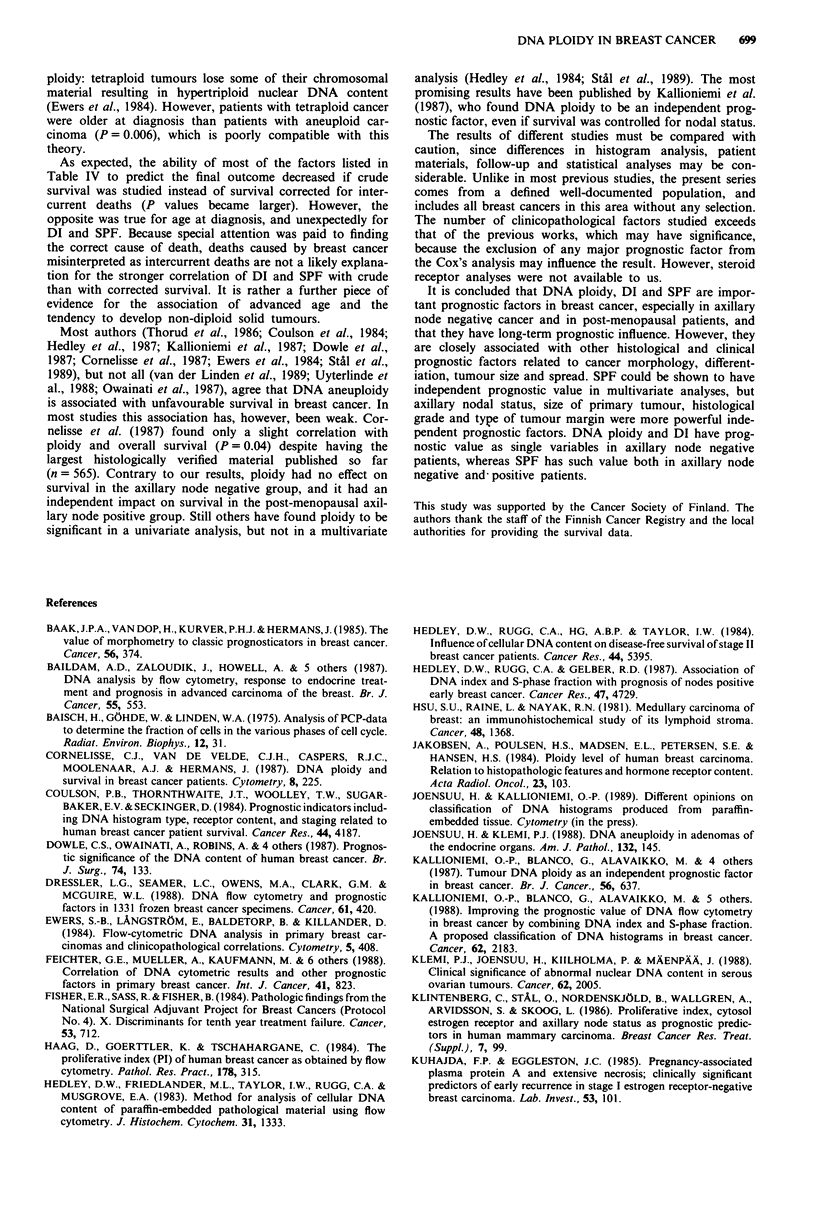

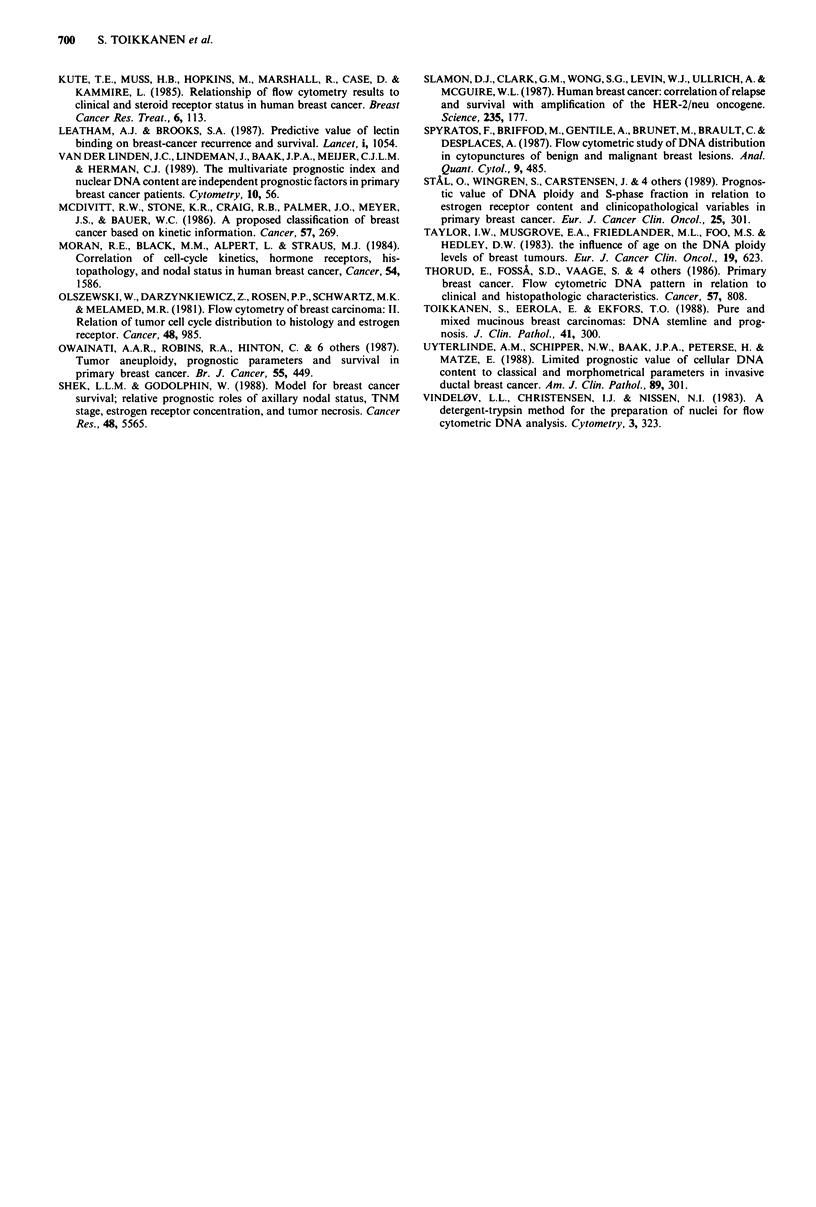


## References

[OCR_01375] Baak J. P., Van Dop H., Kurver P. H., Hermans J. (1985). The value of morphometry to classic prognosticators in breast cancer.. Cancer.

[OCR_01380] Baildam A. D., Zaloudik J., Howell A., Barnes D. M., Turnbull L., Swindell R., Moore M., Sellwood R. A. (1987). DNA analysis by flow cytometry, response to endocrine treatment and prognosis in advanced carcinoma of the breast.. Br J Cancer.

[OCR_01386] Baisch H., Göhde W., Linden W. A. (1975). Analysis of PCP-data to determine the fraction of cells in the various phases of cell cycle.. Radiat Environ Biophys.

[OCR_01391] Cornelisse C. J., van de Velde C. J., Caspers R. J., Moolenaar A. J., Hermans J. (1987). DNA ploidy and survival in breast cancer patients.. Cytometry.

[OCR_01398] Coulson P. B., Thornthwaite J. T., Woolley T. W., Sugarbaker E. V., Seckinger D. (1984). Prognostic indicators including DNA histogram type, receptor content, and staging related to human breast cancer patient survival.. Cancer Res.

[OCR_01402] Dowle C. S., Owainati A., Robins A., Burns K., Ellis I. O., Elston C. W., Blamey R. W. (1987). Prognostic significance of the DNA content of human breast cancer.. Br J Surg.

[OCR_01407] Dressler L. G., Seamer L. C., Owens M. A., Clark G. M., McGuire W. L. (1988). DNA flow cytometry and prognostic factors in 1331 frozen breast cancer specimens.. Cancer.

[OCR_01412] Ewers S. B., Långström E., Baldetorp B., Killander D. (1984). Flow-cytometric DNA analysis in primary breast carcinomas and clinicopathological correlations.. Cytometry.

[OCR_01417] Feichter G. E., Mueller A., Kaufmann M., Haag D., Born I. A., Abel U., Klinga K., Kubli F., Goerttler K. (1988). Correlation of DNA flow cytometric results and other prognostic factors in primary breast cancer.. Int J Cancer.

[OCR_01422] Fisher E. R., Sass R., Fisher B. (1984). Pathologic findings from the National Surgical Adjuvant Project for Breast Cancers (protocol no. 4). X. Discriminants for tenth year treatment failure.. Cancer.

[OCR_01428] Haag D., Goerttler K., Tschahargane C. (1984). The proliferative index (PI) of human breast cancer as obtained by flow cytometry.. Pathol Res Pract.

[OCR_01433] Hedley D. W., Friedlander M. L., Taylor I. W., Rugg C. A., Musgrove E. A. (1983). Method for analysis of cellular DNA content of paraffin-embedded pathological material using flow cytometry.. J Histochem Cytochem.

[OCR_01444] Hedley D. W., Rugg C. A., Gelber R. D. (1987). Association of DNA index and S-phase fraction with prognosis of nodes positive early breast cancer.. Cancer Res.

[OCR_01439] Hedley D. W., Rugg C. A., Ng A. B., Taylor I. W. (1984). Influence of cellular DNA content on disease-free survival of Stage II breast cancer patients.. Cancer Res.

[OCR_01449] Hsu S. M., Raine L., Nayak R. N. (1981). Medullary carcinoma of breast: an immunohistochemical study of its lymphoid stroma.. Cancer.

[OCR_01454] Jakobsen A., Poulsen H. S., Madsen E. L., Petersen S. E., Hansen H. S. (1984). Ploidy level of human breast carcinoma. Relation to histopathologic features and hormone receptor content.. Acta Radiol Oncol.

[OCR_01465] Joensuu H., Klemi P. J. (1988). DNA aneuploidy in adenomas of endocrine organs.. Am J Pathol.

[OCR_01469] Kallioniemi O. P., Blanco G., Alavaikko M., Hietanen T., Mattila J., Lauslahti K., Koivula T. (1987). Tumour DNA ploidy as an independent prognostic factor in breast cancer.. Br J Cancer.

[OCR_01474] Kallioniemi O. P., Blanco G., Alavaikko M., Hietanen T., Mattila J., Lauslahti K., Lehtinen M., Koivula T. (1988). Improving the prognostic value of DNA flow cytometry in breast cancer by combining DNA index and S-phase fraction. A proposed classification of DNA histograms in breast cancer.. Cancer.

[OCR_01481] Klemi P. J., Joensuu H., Kiilholma P., Mäenpä J. (1988). Clinical significance of abnormal nuclear DNA content in serous ovarian tumors.. Cancer.

[OCR_01493] Kuhajda F. P., Eggleston J. C. (1985). Pregnancy-associated plasma protein A and extensive necrosis. Clinically significant predictors of early recurrence in stage I estrogen receptor-negative breast carcinoma.. Lab Invest.

[OCR_01501] Kute T. E., Muss H. B., Hopkins M., Marshall R., Case D., Kammire L. (1985). Relationship of flow cytometry results to clinical and steroid receptor status in human breast cancer.. Breast Cancer Res Treat.

[OCR_01507] Leathem A. J., Brooks S. A. (1987). Predictive value of lectin binding on breast-cancer recurrence and survival.. Lancet.

[OCR_01516] McDivitt R. W., Stone K. R., Craig R. B., Palmer J. O., Meyer J. S., Bauer W. C. (1986). A proposed classification of breast cancer based on kinetic information: derived from a comparison of risk factors in 168 primary operable breast cancers.. Cancer.

[OCR_01521] Moran R. E., Black M. M., Alpert L., Straus M. J. (1984). Correlation of cell-cycle kinetics, hormone receptors, histopathology, and nodal status in human breast cancer.. Cancer.

[OCR_01527] Olszewski W., Darzynkiewicz Z., Rosen P. P., Schwartz M. K., Melamed M. R. (1981). Flow cytometry of breast carcinoma: II. Relation of tumor cell cycle distribution to histology and estrogen receptor.. Cancer.

[OCR_01533] Owainati A. A., Robins R. A., Hinton C., Ellis I. O., Dowle C. S., Ferry B., Elston C. W., Blamey R. W., Baldwin R. W. (1987). Tumour aneuploidy, prognostic parameters and survival in primary breast cancer.. Br J Cancer.

[OCR_01538] Shek L. L., Godolphin W. (1988). Model for breast cancer survival: relative prognostic roles of axillary nodal status, TNM stage, estrogen receptor concentration, and tumor necrosis.. Cancer Res.

[OCR_01544] Slamon D. J., Clark G. M., Wong S. G., Levin W. J., Ullrich A., McGuire W. L. (1987). Human breast cancer: correlation of relapse and survival with amplification of the HER-2/neu oncogene.. Science.

[OCR_01550] Spyratos F., Briffod M., Gentile A., Brunet M., Brault C., Desplaces A. (1987). Flow cytometric study of DNA distribution in cytopunctures of benign and malignant breast lesions.. Anal Quant Cytol Histol.

[OCR_01556] Stål O., Wingren S., Carstensen J., Rutqvist L. E., Skoog L., Klintenberg C., Nordenskjöld B. (1989). Prognostic value of DNA ploidy and S-phase fraction in relation to estrogen receptor content and clinicopathological variables in primary breast cancer.. Eur J Cancer Clin Oncol.

[OCR_01562] Taylor I. W., Musgrove E. A., Friedlander M. L., Foo M. S., Hedley D. W. (1983). The influence of age on the DNA ploidy levels of breast tumours.. Eur J Cancer Clin Oncol.

[OCR_01567] Thorud E., Fosså S. D., Vaage S., Kaalhus O., Knudsen O. S., Børmer O., Shoaib M. C. (1986). Primary breast cancer. Flow cytometric DNA pattern in relation to clinical and histopathologic characteristics.. Cancer.

[OCR_01572] Toikkanen S., Eerola E., Ekfors T. O. (1988). Pure and mixed mucinous breast carcinomas: DNA stemline and prognosis.. J Clin Pathol.

[OCR_01577] Uyterlinde A. M., Schipper N. W., Baak J. P., Peterse H., Matze E. (1988). Limited prognostic value of cellular DNA content to classical and morphometrical parameters in invasive ductal breast cancer.. Am J Clin Pathol.

[OCR_01583] Vindeløv L. L., Christensen I. J., Nissen N. I. (1983). A detergent-trypsin method for the preparation of nuclei for flow cytometric DNA analysis.. Cytometry.

[OCR_01510] van der Linden J. C., Lindeman J., Baak J. P., Meijer C. J., Herman C. J. (1989). The Multivariate Prognostic Index and nuclear DNA content are independent prognostic factors in primary breast cancer patients.. Cytometry.

